# Neocortical activity is stimulus- and scale-invariant

**DOI:** 10.1371/journal.pone.0177396

**Published:** 2017-05-10

**Authors:** Yahya Karimipanah, Zhengyu Ma, Jae-eun Kang Miller, Rafael Yuste, Ralf Wessel

**Affiliations:** 1 Department of Physics, Washington University, St. Louis, Missouri, United States; 2 Neurotechnology Center and Department of Biological Sciences, Columbia University, New York, New York, United States; University of Michigan, UNITED STATES

## Abstract

Mounting evidence supports the hypothesis that the cortex operates near a critical state, defined as the transition point between order (large-scale activity) and disorder (small-scale activity). This criticality is manifested by power law distribution of the size and duration of spontaneous cascades of activity, which are referred as *neuronal avalanches*. The existence of such neuronal avalanches has been confirmed by several studies both in vitro and in vivo, among different species and across multiple spatial scales. However, despite the prevalence of scale free activity, still very little is known concerning whether and how the scale-free nature of cortical activity is altered during external stimulation. To address this question, we performed in vivo two-photon population calcium imaging of layer 2/3 neurons in primary visual cortex of behaving mice during visual stimulation and conducted statistical analyses on the inferred spike trains. Our investigation for each mouse and condition revealed power law distributed neuronal avalanches, and irregular spiking individual neurons. Importantly, both the avalanche and the spike train properties remained largely unchanged for different stimuli, while the cross-correlation structure varied with stimuli. Our results establish that microcircuits in the visual cortex operate near the critical regime, while rearranging functional connectivity in response to varying sensory inputs.

## Introduction

**Fig 1:**
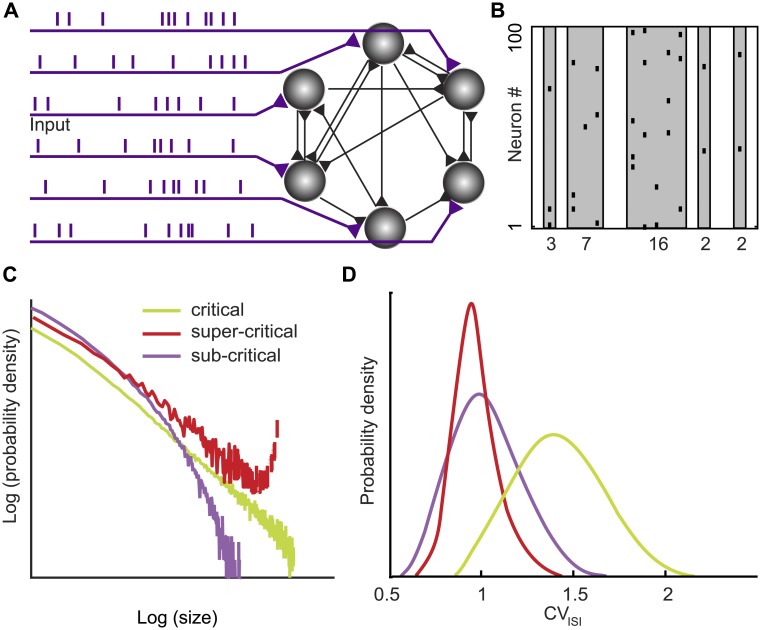
A quantification of the level of spike correlation in recurrent cortical circuits and its variation with external inputs is essential for understanding cortical computation. (A) Schematic of a recurrent neural circuit consisting of neurons (gray), connections (black), and external inputs (purple). (B) Neuronal avalanches (gray) are contiguous bouts of spikes (black raster) across the recorded neurons. The spike count within an avalanche determines the avalanche size (numbers). (C) The shape of the avalanche size distribution reflects the level of spatiotemporal correlation within the network. A power law avalanche size distribution (straight line in the log log plot) is a characteristic feature of the critical (green) network state. Deviations from the power law distributions indicate the subcritical (purple) and supercritical (red) network state. (D) Theory predicts that operating near the critical network state impacts the variability of neuronal spiking as characterized by the coefficient of variation (CV_ISI_) of the interspike interval (ISI). Specifically, irregular spiking (CV_ISI_ > 1) has been predicted to emerge at the critical network (green), whereas for the subcritical (purple) and the supercritical (red) network state the CV_ISI_ distribution peaks near 1.

How does the activity of individual neurons and neuronal circuits give rise to knowledge representation, computation, and cognition? This fundamental question in neuroscience [1] is deeply confounded by the recurrent nature of cortical circuits [2,3] (Fig 1A), which shows its dynamic face (Fig 1B) in intrinsically generated cortical activity [4–6]. Specifically, it has long been argued that recurrent cortical circuits self-organize towards a dynamical critical regime [7]. Such critical network state straddles the boundary between two distinct regimes of order and disorder [8]. The hypothesized critical dynamics, at the boundary between the two regimes, is predicted to reveal itself in the scale-free neuronal activity [9] (Fig 1C) and in the irregular nature of neuronal spiking [10] (Fig 1D).

The self-organized criticality hypothesis for the nervous system [11,12] continues to garner support from large-scale measurements of integrated neural activity in intact brains ranging from local field potential recordings in reptiles [13] to magnetoencephalography in humans [14]. However, whether the natural small-scale building blocks of the brain, the neurons, self-organize into microcircuits operating near the critical regime during sensory processing continues to remain a crucial question in neuroscience [15,16].

Here, we addressed this question by imaging the activity from populations of layer 2/3 neurons in primary visual cortex of awake and behaving mice during visual stimulation. We analyzed the spike trains with respect to (i) the statistics of the spatiotemporal cascades of activity (neuronal avalanches) (Fig 1B and 1C), (ii) the statistical properties of individual spike trains (Fig 1D), and (iii) the pairwise correlation of spike trains. Results from this combination of analysis tools support the notion that neocortical microcircuits operate near criticality independent of stimulus condition, while rearranging correlation patterns in a stimulus specific manner.

## Materials and methods

### Animals

This study was carried out in strict accordance with the recommendations in the Guide for the Care and Use of Laboratory Animals of the National Institutes of Health. The protocol was approved by the Committee on the Ethics of Animal Experiments of the Columbia University (Permit Number: AC-AAAD0720). All surgery was performed under isoflurane anesthesia, and all efforts were made to minimize suffering. This study represents an independent analysis of experiments described previously [17]. In brief, experiments were performed on C57BL/6 mice (n = 6) or on parvalbumin-Cre (n = 2) or somatostatin-Cre (n = 2) × LSL-tdTomato transgenic mice, obtained from The Jackson Laboratory, at the age of postnatal day (P) P40–80 [18–20]. Six mice were used for awake preparation, and four mice were used for anesthetized preparation. During surgery, mice were anesthetized with isoflurane (initially 2% (partial pressure in air) and reduced to 1%). A small circle (1–2 mm in diameter) was thinned over the left V1 using a dental drill to mark the site for craniotomy (centered at 2.5 mm lateral from the lambda, putative monocular region). A titanium head plate was attached to the skull using dental cement. Mice underwent training to maneuver on a spherical treadmill with their head fixed for 1–3 h each day for 2–3 d.

All animals were monitored daily, including weekend days, according to an internal schedule. During daily monitoring if an animal was extremely jumpy to touch, vocalized while undisturbed in cage, was unable to groom, or showed lethargy, ruffled fur, or signs of abnormal nursing behavior, we further determined distress or pain by comparing weight loss to other age and sex matched animals. A couple of animals became ill after surgery, were euthanized, and were not included in this study. All mice used in this study were healthy throughout the experiment. Infection at site of surgery confounds experimental results making any data acquired invalid. Thus, animals with infection were euthanized to prevent further pain. A couple of mice became ill and were euthanized. They were not included in the study. No animals died prior to the experimental endpoint. Daily monitoring of every injected animal was carried out by our laboratory, including weekend days, according to an internal schedule. In addition, weight loss compared to baseline pre-surgery was monitored to assess pain and distress. Animals were weighted twice weekly and euthanized if weight loss was greater than 20%. Animals were euthanized by carbon dioxide to prevent further pain. The secondary method of euthanasia was cervical dislocation.

### Imaging

On the imaging day, mice were anesthetized with isoflurane and the craniotomy, marked previously, was completed for dye injection. For bulk loading of cortical neurons Oregon Green Bapta-1 AM (Molecular Probes) was first dissolved in 4 μL of freshly prepared DMSO containing 20% Pluronic F-127 (Molecular Probes) and then further diluted in 35 μL of dye buffer [150 mM NaCl, 2.5 mM KCl, and 10 mM Hepes (pH 7.4) [21]. Sulforhodamine 101 (50 μM; Molecular Probes) was added to the solution to label astrocytes [22]. The dye was slowly pressure-injected into the left visual cortex at a depth of 150–200 μm at an angle of 30° with a micropipette (4–7 MΩ, 10 psi, 8 min) under visual control by two—photon imaging (20x water immersion objective, 0.5 N.A.; Olympus). The activity of cortical cells was recorded by imaging fluorescence changes with a two-photon microscope (Moveable Objective Microscope; Sutter Instrument) and a Ti:sapphire laser (Chameleon Vision II; Coherent) at 880 nm or 1,040 nm through a 20x (0.95 N.A.; Olympus) or 25x (1.05 N.A.; Olympus) water immersion objective. Scanning and image acquisition were controlled by Sutter software (4.07 frames per second for 512 × 512 pixels, Mscan; Sutter Instrument).

### Visual stimulation

Visual stimuli were generated using the MATLAB (MathWorks) Psychophysics Toolbox [23] and displayed on a liquid crystal display monitor (19-inch diameter, 60-Hz refresh rate) positioned 15 cm from the right eye, roughly at 45° to the long axis of the animal. Spontaneous calcium signals were measured for ~13 min in the dark at the beginning of the experiments and sometimes in the middle of the experiments (with a monitor and room lights turned off). The imaging setup was completely enclosed with blackout fabric (Thorlabs). After spontaneous calcium signals were collected, mice were presented with either sequences of full-field grating stimuli or a natural movie (the order of presentations was alternated randomly). Square or sine wave gratings (100% contrast, 0.035 cycles per degree, two cycles per second) drifting in eight different directions in random order were presented for 5 s, followed by 5 s of mean luminescence gray screen (10 repetitions). A natural movie (Moose in the Glen, from the British Broadcasting Corporation’s Natural World documentary series) consisting of 10 distinct natural scenes in 30-s sequences was played using the MATLAB Psychophysics Toolbox (20 repetitions). In some experiments, a natural movie was played using the QuickTime Player (Apple). The visual responsiveness of the recorded neurons has been described previously [17]. The sequences of gratings or a natural movie stimulation played in MATLAB were synchronized with image acquisition using Sutter software (Mscan; Sutter Instrument). Locomotion of a mouse was not associated with motion of the visual scene relative to the mouse.

### Image analysis

**Fig 2:**
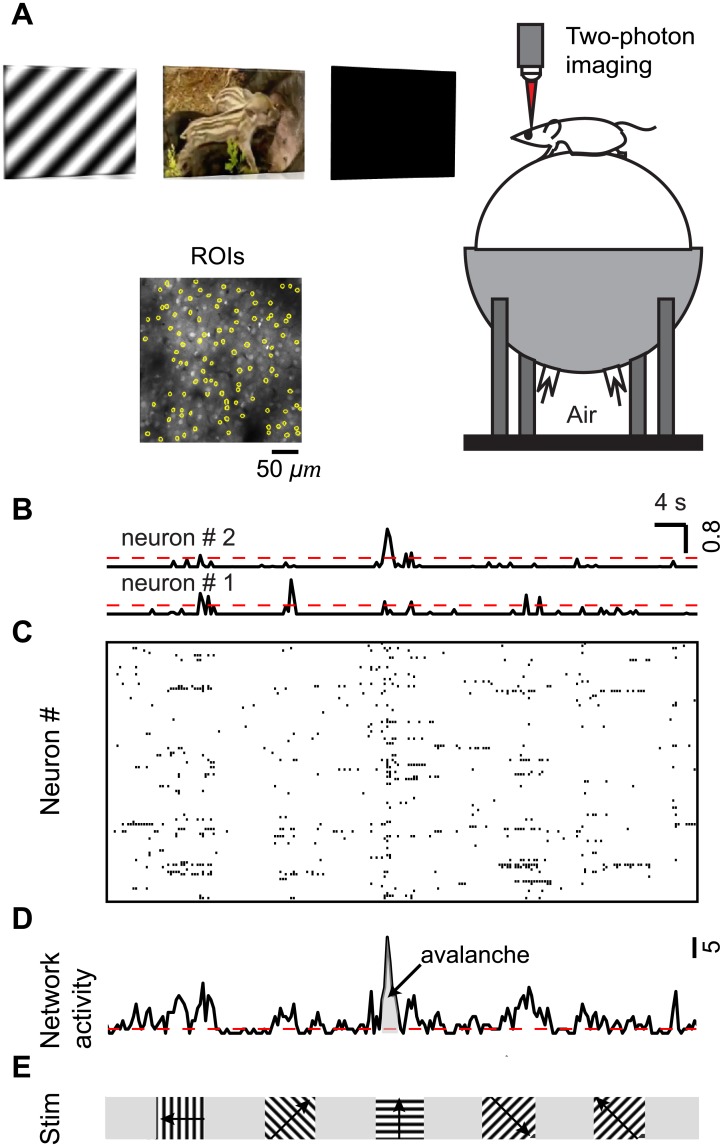
Quantifying the level of coordination at cellular resolution within cortical microcircuits of behaving mice. (A) Illustration of population Ca imaging from the visual cortex in a head-fixed awake mouse on a spherical treadmill during viewing of three types of visual stimuli: square or sine wave grating, natural movie, and dark screen. For clarity, the head fixation was omitted from the drawing. Inset: Two-photon microscopic image of a typical field of view from neurons bolus-loaded with Oregon Green Bapta-1 AM (OGB- 1) dye in layer 2/3 of V1. Regions of interest (ROIs, yellow) are overlaid on the image. (B) Inferred spike probability for two representative neurons during visual stimulation with sine-wave gratings (see (E)). Spike probability was inferred from calcium sensitive dye fluorescent signals using a spike inference algorithm (Methods). Spike probability was then thresholded (dashed red line) to a level of 3 SDs above 0, and converted to 1 (active) or 0 (inactive). (C) Raster plot of activity (moving gratings) constructed using the thresholded spike probability. Each row represents a single neuron, and each mark represents the inferred spiking activity of that neuron, i.e., the thresholded spike probability with value 1 (active). (D) “Network activity” (black) is the sum of all spiking neurons in a time bin (250 ms), i.e., the thresholded inferred spike probability summed over all recorded neurons in that time bin. A threshold (dashed red line) at median network activity defined the start and end of a “neuronal avalanche” as the time points of crossing this threshold. The avalanche size (gray) is the integrated network activity for the avalanche duration, i.e., the time between threshold crossings. (E) For the data in (B) to (D), moving gratings (100% contrast, 0.035 cycles per degree, two cycles per second) drifting in eight different directions in random order were presented for 5 s, followed by 5 s of mean luminescence gray screen.

The raw images were processed to correct brain motion artifacts using the enhanced correlation coefficient image alignment algorithm [24] or a hidden Markov model implemented previously [25,26]. Initial image processing was carried out using custom-written software in MATLAB (Caltracer 2.5, available at our laboratory website). Cell outlines were detected using an automated algorithm based on fluorescence intensity, cell size, and cell shape, and were adjusted by visual inspection. Cell-based regions of interest (ROIs) were then shrunk by 5–10% to minimize the influence of the neuropil signal around the cell bodies (Fig 2A). All pixels within each ROI were averaged to give a single time course, and ΔF/F was calculated by subtracting each value with the mean of the lower 50% of previous 10-s values and dividing it by the mean of the lower 50% of previous 10-s values. Neurons with noisy signal with no apparent calcium transient for a given stimulus condition were detected by visual inspection and excluded from further analysis for that stimulus condition. Spike probability was inferred from calcium signals using a fast, non-negative deconvolution method [27]. Briefly, the baseline of calcium signals was detrended, and ΔF/F was then calculated before applying an algorithm to infer spike probability. The decay constant of calcium transients was set to 0.8 s. The output was normalized by a maximum value in each neuron. Spike probability was then thresholded to a level of 3 SDs above 0, determined from spike probabilities of the entire population in each experiment (Fig 2B). The values above a threshold were set to 1, and the values below a threshold were set to 0. These binary activity data were then used for subsequent analyses unless otherwise indicated. Although most spikes resulted in significant somatic calcium transients with a calcium indicator and analysis threshold similar to our experiments [28], we likely underestimated the presence of action potentials, particularly when neurons fire a single action potential or at frequencies higher than 40 Hz [29].

### Spike train analysis

From the thresholded inferred spike probability of the recorded neurons (Fig 2C), we obtained the “network activity” (Fig 2D) as the sum of all spiking neurons in a time bin (250 ms), i.e., the thresholded inferred spike probability summed over all recorded neurons (Fig 2D). Based on the network activity, we defined a “neuronal avalanche” by introducing a threshold at median network activity [30,31]. This definition of a neuronal avalanche is well suited to recordings from a large population of neurons, which yields few or no silent periods in the recorded population activity. For completeness, we note that this definition is blind to the spatial location of the neurons. An avalanche starts when the network activity crosses the threshold from below and ends when the network activity crosses the threshold from above. We quantified each neuronal avalanche by its size *S*, i.e., the integrated network activity between threshold crossings, and its duration *D*, i.e., the time between threshold crossings (Fig 2D). Avalanches were analyzed separately for the three different conditions of visual stimulation.

Using maximum likelihood estimation methods, we fitted a truncated power law $f\left(S\right)=\frac{S^{-\tau }}{\sum_{Smin}^{Smax}S^{-\tau }}$ to the avalanche size distribution of *N*_*av*_ avalanches using the following iterative procedure. (i) The maximum avalanche size *S*_*max*_ was taken as the largest observed avalanche size. (ii) The exponent τ was estimated for three values of the minimum avalanche size *S*_*min*_ ranging from 1 to 3 and the corresponding Kolmogorov-Smirnov (KS) values were obtained. (iii) The minimum avalanche size *S*_*min*_ and the corresponding exponent τ yielding the smallest KS value were chosen. (iv) When $KS\;<\;1/\sqrt{N_{av}}$, the exponent estimation was completed. Otherwise, the procedure (ii) to (iv) was repeated with the maximum avalanche size *S*_*max*_ reduced by 1 until the condition $KS\;<\;1/\sqrt{N_{av}}$, was satisfied. We applied the same fitting procedure to the avalanche duration distributions.

To evaluate whether a power law was a plausible fit of an avalanche distribution, we performed hypothesis testing. We simulated 1000 artificial power law distributions (surrogate distributions) with the same exponent, number of avalanches, minimum avalanche size, and maximum avalanche size, as estimated from the experimental avalanche distribution. Specifically, using the inverse method, the surrogate distributions were generated according to *S* = *S*_*min*_(1 − *r*)^−1/(*τ* − 1)^ where *r* was a random number drawn from a uniform distribution between 0 and 1. Thereafter, the distribution was upper-truncated by setting a cut-off at the maximum value observed in the empirical data *S*_*max*_. This procedure worked well for generating avalanches of *S*_*min*_ larger than 1. For *S*_*min*_ = 1, we used an alternative acceptance-rejection method [32].

The deviation between the simulated surrogate distributions and a perfect power law was quantified with the *KS* statistics. The *p* value was calculated as the fraction of the surrogate distributions with *KS* values smaller than the *KS* value of the corresponding experimental avalanche distribution. We took the significance level to be 0.05, i.e., for *p* < 0.05 the power law hypothesis was rejected, whereas for *p* ≥ 0.05 the power law hypothesis was not rejected. This hypothesis testing procedure is illustrated, by plotting the experimental avalanche distribution over a gray band that delineates the 5 to 95 percentiles of the surrogate distributions.

The uncertainty of the exponent estimation was computed using bootstrap method. After estimating the exponent from the experimental avalanche distribution, we resampled actual avalanches (with replacement) 1000 times and then fitted the resampled data to a power law and estimated the exponent. The standard deviation of re-estimated exponents provided an estimate of the uncertainty in the exponent estimation from the experimental avalanche distribution.

To test whether average avalanche size scales with duration according to < *S* > ~ *D*^*β*^, we estimated the fitted *β* from the experimental data using linear regression. We then compared the fitted *β* to the predicted *β*, where the predicted *β* = (*α* − 1)/(*τ* − 1) was obtained from the size and duration exponents derived from pure power law estimation [33].

Cross-correlation coefficients were obtained by calculating the zero-lag pairwise Pearson correlation coefficients of the thresholded inferred spike probability for a given stimulus condition using the Matlab corrcoef routine. The resulting matrix of cross-correlation coefficients for grating stimulation was clustered using hierarchical clustering with maximum or complete-linkage clustering (Matlab dendrogram routine). Neurons were rearranged accordingly to visualize the clusters in the cross-correlation matrix during grating stimulation. The clusters seen for grating stimulation indicate groups of neurons with similar orientation selectivity. Subsequently, the cross-correlation matrices for ongoing activity and natural movie stimulation were plotted without rearranging the neurons. This display shows the reorganization of the cross-correlation when varying the stimulus condition.

## Results

To record neuronal population spiking at cellular resolution within cortical microcircuits, we performed two-photon population calcium imaging of layer 2/3 neurons in primary visual cortex of head-fixed awake and behaving mice during three conditions of visual stimulation: dark screen, moving grating, and naturalistic movie (Fig 2A). Recordings taken under the dark screen condition were interpreted as ongoing activity. For each mouse and stimulus condition we obtained the inferred spike trains from some 100 closely-spaced neurons for 13 minutes (Fig 2B and 2C).

### Cortical microcircuits operate near criticality during three stimulus conditions

We analyzed the spike trains employing the concept of “neuronal avalanches” [9], which are bouts of elevated network activity, revealing correlations both among neurons and in time. Specifically, we defined a neuronal avalanche based on the threshold crossing of the network activity [30,31]. We quantified each neuronal avalanche by its size *S*, i.e., the number of spikes, quantified by the area under the curve between threshold crossings, and its duration *D*, i.e., the time between threshold crossings (Fig 2D and 2E).

**Fig 3:**
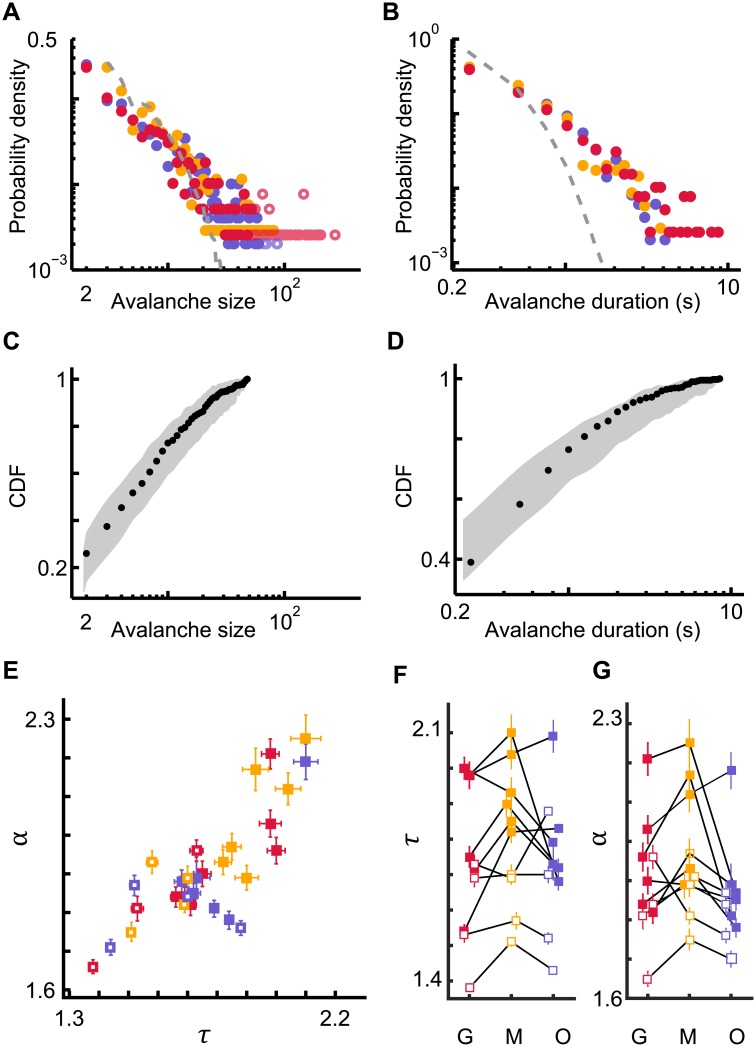
Avalanche size and duration distributions of cortical spiking activity for three different conditions of visual stimulation. (A, B) Probability density functions for avalanche sizes and durations for the three stimulus conditions: grating (red), movie (yellow), and ongoing (blue). The solid dots denote the avalanches included for fitting to a truncated power law; open circles denote avalanches that were excluded in the fitting procedure (see Materials and methods). Shuffling spike times abolishes large avalanches and results in an avalanche size distribution (dashed gray lines) that is inconsistent with a power law. (C, D) Cumulative probability density function (CDF, black dots) of the experimental data from A and B. For visual comparison, the gray shading indicates the range (5–95%) of expected probabilities for the truncated power law with the same exponent as estimated from the experimental data and with the same number of samples. (E) Estimated avalanche size (τ) and duration (α) exponents for all mice and stimulus conditions for which the avalanche distributions fit power laws according to strict statistical criteria. (F, G) Estimated avalanche size (τ) and duration (α) exponents for the three stimulus conditions: grating (G), movie (M), and ongoing (O). Exponents from the same mouse are connected by black lines.

Avalanches were diverse in spatiotemporal scale. Specifically, both avalanche size and duration distributions were typically consistent with power laws, *P*(*S*)~*S*^−*τ*^ (Fig 3A and S1–S3 Figs) and *P*(*D*)~*D*^−*α*^ (Fig 3B and S4–S6 Figs). The closeness of the avalanche distributions to power laws was evaluated using rigorous statistical criteria (Method, Fig 3C and 3D). Avalanche size and duration distributions were typically power-law distributed for ten mice and three types of visual stimulus conditions (dark screen, moving grating, naturalistic movie; n = 30 data sets). Notably, randomizing the inferred spike times abolished the power-law distributions of avalanche size and duration (Fig 3A and S2 and S5 Figs).

Importantly, despite the different spatiotemporal structure of the three stimulus conditions, recorded population activities typically resulted in power-law avalanche distributions (Fig 3A and 3B), indicating that the scale-free nature of cortical activity was due to inherent microcircuit dynamics rather than externally imposed stimulus statistics. While power-law avalanche distributions were largely robust for all data sets (S1 and S4 Figs), the exponent values covered a broad range (Fig 3E). The range of values is in line with earlier experimental results [9,13,34,35] and, in computational studies of a critical model network, has been demonstrated to result from subsampling [13].

In addition, the exponent values tended to be smaller for anesthetized mice compared to awake mice (Fig 3E). This observation implies extended spatiotemporal correlations in the population activity of the anesthetized mice, thus yielding neuronal avalanches of larger sizes and longer duration.

Concerning the potential impact of the stimulus condition on the exponent values, the data were less conclusive (Fig 3F and 3G). While for many mice the exponent values varied for different stimulus conditions, the direction of change was not consistent from mouse to mouse. When considering the population of mice, only the size exponent for the movie stimulation was significantly larger than the size exponent for the grating stimulation (p = 0.034, t-test).

**Fig 4:**
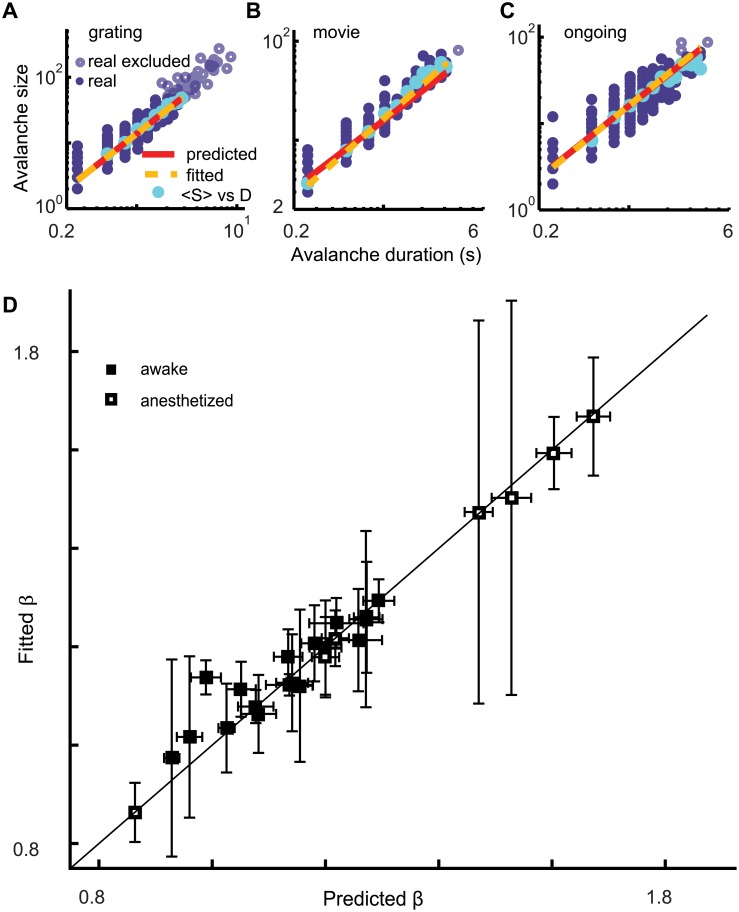
Average avalanche size scales with duration as predicted for a network operating near criticality. (A-C) For each avalanche (solid purple dots) the size is plotted (log-log scale) vs the duration for the three stimulus conditions (mouse #3). For each avalanche duration the average avalanche size (cyan dots) is plotted. The linear relationship on logarithmic axes reveals a power law relationship < *S* > ~ *D*^*β*^ between average avalanche size and duration as predicted by criticality theory. The fitted exponent β is derived from the linear regression line (yellow dashes). The predicted line (red) is derived from the predicted exponent *β* = (*α* − 1)/(*τ* − 1). Solid purple dots were included in the exponent estimation, open circles were not (see Materials and methods). (D) The summary plot for all mice shows that the fitted exponent *β* largely matches the predicted exponent *β* = (*α* − 1)/(*τ* − 1) for all mice and stimulus conditions. This is despite the fact that different values were found for size exponents *τ* and duration exponents *α* for different mice and stimulus conditions.

Power laws provide necessary, but insufficient evidence for critical dynamics [36]. Additional tests are needed to determine whether criticality underlies the experimentally observed power laws. Two such tests arise from a particular relationship between the size and duration of avalanches, which is predicted to occur at criticality [37,38]. First, the average avalanche size scales with duration according to < *S* > ~ *D*^*β*^. Second, the exponent *β* is not independent, but rather depends on the exponents τ and α according to *β* = (*α* − 1)/(*τ* − 1). Our experiments confirmed both these predictions from the scaling relation for all stimulus conditions. Average avalanche size scaled with duration < *S* > ~ *D*^*β*^ according to a power law (Fig 4A–4C and S7–S9 Figs) and the observed values of *τ* and α provided a good prediction *β* = (*α* − 1)/(*τ* − 1) of the fitted *β* (Fig 4D). The observations of power laws (Fig 3) and scaling relations (Fig 4) together provide strong evidence that the inspected cortical microcircuits operate near criticality independent of the stimulus condition.

### Irregular single-neuron spiking is consistent with network criticality

**Fig 5:**
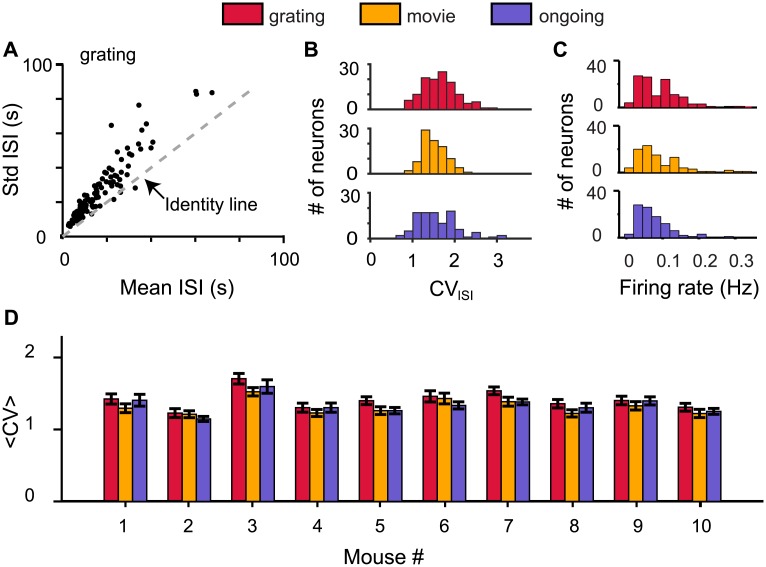
Irregular spiking at network criticality. (A) The standard deviation (Std) of the inter spike intervals (ISIs) was larger than the mean ISI for most neurons (black dots) for mouse #3 (grating). (B) The ISI coefficient of variation (CV_ISI_) distributions for the recorded neurons from mouse #3 under three stimulus conditions. (C) The firing rate distributions for the recorded neurons from mouse #3 under three stimulus conditions. (D) The average coefficient of variation 〈*CV*〉 (mean ± SEM) for all 10 mice and three stimulus conditions tested.

Complementary to the avalanche analysis, the coefficient of variation (CV_ISI_), defined as the ratio of the standard deviation and the mean of the inter spike intervals (ISI) of individual spike trains, provides qualitatively different measures to test the criticality hypothesis. Irregular spiking with a CV_ISI_ distribution with peak above 1 is predicted to be an emergent property of a neural network operating near criticality [10]. Our experiments give credence to this prediction concerning the statistical properties of spike trains. The standard deviation (Std) of the ISIs was larger than the mean ISI for most neurons (Fig 5A). This translated into a CV_ISI_ distribution peaked above CV_ISI_ values of 1 (Fig 5B and S10 Fig). In addition, the firing rate distribution was right skewed for all conditions (Fig 5C). For the ten mice and three types of visual stimulus conditions the 〈*CV*〉 was significantly larger than 1 and for a given mouse the 〈*CV*〉 values were similar for different stimulus conditions (Fig 5D).

### Stimulus condition redistributes spatiotemporal activity

**Fig 6:**
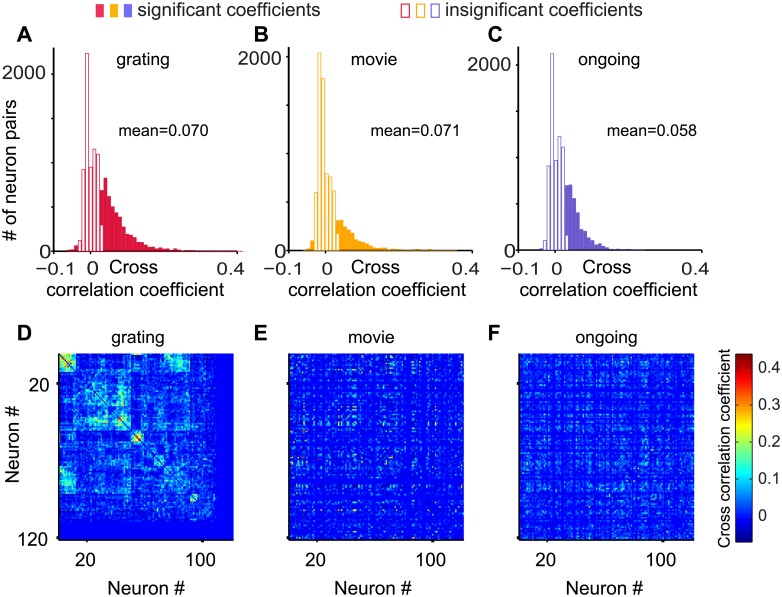
The cross-correlation coefficients among the pairs of neurons rearrange for different stimulus conditions. (A-C) The distributions of the zero-lag pairwise Pearson cross-correlation coefficients of the thresholded inferred spike probabilities for the three stimulus conditions. Significance testing was obtained by comparing with uncorrelated spike trains of the same mean rate and adopting a p-value threshold of 0.1. (D) The cross-correlation coefficient matrix for the grating stimulus, with the matrix clustered using a hierarchical clustering algorithm (see Materials and methods). A subset of rows/columns are blank because neurons with noisy signal with no apparent calcium transient for a give stimulus condition were detected by visual inspection and excluded from further analysis for that stimulus condition. (E, F) The cross-correlation coefficient matrix for the other two stimulus conditions (movie, ongoing), while maintaining the order of neurons as in (D). This display illustrates the reorganization of the cross-correlation when varying the stimulus condition.

In summary, we observed that the statistical measures of both spatiotemporal activity (Figs 3 and 4) and the spike train properties (Fig 5) were largely stimulus invariant. This observation raised the question whether stimulus condition impacted other measures of spatiotemporal activity as might be expected for knowledge representation in a neural network. One such measure of spatiotemporal activity is the pairwise cross-correlation coefficients. Our experiments showed that cross-correlation coefficients were broadly distributed for all stimuli tested (Fig 6A–6C and S11 Fig). Importantly, changing the stimulus condition caused a redistribution of the pairwise cross-correlation coefficients among the pairs (Fig 6D–6F and S12 Fig).

## Discussion

Our results support the hypothesis that cortical microcircuits at cellular resolution operate near criticality during sensory processing, while rearranging functional connectivity in response to varying sensory inputs.

Ongoing innovations in imaging technology permit the probing of neural spiking of ever larger number of neurons [39,40,41]. By extending the evidence for the criticality hypothesis to the cellular resolution of cortical microcircuits, we advanced our understanding of cortical dynamics in a fundamental manner. The testimony shown here (Figs 3 and 4) transcends beyond the coarse spatial scale of previous tests of criticality in cerebral cortex of awake subjects, which employed recordings of ongoing integrated large-scale neural activity [42–45]. Building on these important studies of macroscopic cortical activity in brain volumes consisting of thousands to millions of neurons, our results demonstrate that the principle of self-organized criticality applies down to the cellular resolution of some hundred neurons within a microcircuit embedded in the mouse visual cortex.

Importantly, the investigation at cellular resolution addressed a long-standing puzzle of irregular spiking [46] and concurrently pushed open a new window of evidence for the criticality hypothesis of cerebral cortex [10]. Cortical neuron spiking tends to be more irregular than what is expected for a Poisson process; the coefficient of variations (CV) of the inter spike intervals (ISI) are distributed with a peak above one [47]. It has recently been predicted that this statistics of single-neuron spike trains emerges as the property of a recurrent neural network operating near criticality [10]. By measuring the spiking properties of neurons (Fig 5) and evaluating evidence for criticality for the same group of neurons (Figs 3 and 4), we show that irregular spiking (CV > 1) in mouse visual cortex coincides with the cortical circuit operating near criticality. We also show that both the irregularity of spike trains and power law statistics of avalanches are robust with respect to external stimuli.

The observed stimulus invariance of network criticality represents an important advance in systems neuroscience. Much of the interest in the criticality hypothesis [7,12,48] for sensory cortices is fueled by the prediction that the critical regime optimizes aspects of signal processing [49,50]. But do neural networks operate near criticality when external sensory inputs trigger part of the activity? This question, whether neurons self-organize into microcircuits operating near the critical regime during varying external sensory inputs, represents a timely and crucial inquiry in neuroscience [17,51]. Theoretical work suggests that synaptic depression can cause self-organized criticality in weakly driven neural networks [52,53]. Experiments, using local field potential recordings from visual cortex during visual stimulation, seem to support this prediction [13]. In separate studies, it has long been appreciated that the recurrent nature of cortical circuits results in intrinsically generated cortical activity [54, 55] and network states [56], which appear to be modulated, rather than triggered, by sensory inputs [57], while leaving the correlation statistics largely unchanged [17,58,59].

Here we expand on these important earlier studies on the stimulus invariance of certain aspects of neural activity in significant ways. First, we evaluate neural activity at cellular resolution in the visual cortex of awake mice for three different stimulus conditions. Second, we evaluate simultaneously the statistical measures of spike trains (Fig 5), pairwise cross-correlation coefficients (Fig 6A–6C), and spatiotemporal population activity (Figs 3 and 4). Third, we interpret these statistical measures in the context of the network state, namely criticality. In conclusion, our results extend the observation of stimulus invariance of the statistical measures of cortical activity to the level of microcircuits and network state and indicate that different stimuli trigger the redistribution of the functional connectivity matrix (Fig 6D–6F), while maintaining the network state near criticality.

## Supporting information

S1 Fig Avalanche size distributions for all ten mice for three different visual stimuli. Probability density functions for avalanche sizes for each mouse and for the three stimulus conditions: grating (red), movie (yellow), and ongoing (blue). The solid dots denote the avalanches included for fitting to a truncated power law; open circles denote avalanches that were excluded in the fitting procedure (see Methods). P-values of truncated power law estimations (see Methods) are shown for each stimulus condition (color assignment as in legend). We took the significance level to be 0.05, i.e., for *p* < 0.05 the power law hypothesis was rejected, whereas for *p* ≥ 0.05 the power law hypothesis was not rejected.
(PDF)

S2 Fig Comparing avalanche size distributions that were derived from recorded spike trains with those derived from shuffled spike trains. The violet dots denote the avalanches (based on recorded spike trains) that were included for fitting to a truncated power law; pale green dots denote avalanches that were excluded in the fitting procedure (see Methods). Shuffling spike times abolishes large avalanches and results in avalanche size distributions (dashed gray lines) that are inconsistent with a power law.
(PDF)

S3 Fig Cumulative probability density functions (CDF, black dots) of the avalanche size distributions. For visual comparison, the gray shading indicates the range (5–95%) of expected probabilities for the truncated power law with the same exponent as estimated from the experimental data and with the same number of samples (see Methods). P-values of truncated power law estimations (see Methods) are shown for each stimulus condition. We took the significance level to be 0.05, i.e., for *p* < 0.05 the power law hypothesis was rejected, whereas for *p* ≥ 0.05 the power law hypothesis was not rejected.
(PDF)

S4 Fig Avalanche duration distributions for all ten mice for three different visual stimuli. Probability density functions for avalanche durations for each mouse and for the three stimulus conditions: grating (red), movie (yellow), and ongoing (blue). The solid dots denote the avalanches included for fitting to a truncated power law; open circles denote avalanches that were excluded in the fitting procedure (see Methods). P-values of truncated power law estimations (see Methods) are shown for each stimulus condition (color assignment as in legend). We took the significance level to be 0.05, i.e., for *p* < 0.05 the power law hypothesis was rejected, whereas for *p* ≥ 0.05 the power law hypothesis was not rejected.
(PDF)

S5 Fig Comparing avalanche duration distributions that were derived from recorded spike trains with those derived from shuffled spike trains. The violet dots denote the avalanches (based on recorded spike trains) that were included for fitting to a truncated power law; pale green dots denote avalanches that were excluded in the fitting procedure (see Methods). Shuffling spike times abolishes long duration avalanches and results in avalanche duration distributions (dashed gray lines) that are inconsistent with a power law.
(PDF)

S6 Fig Cumulative probability density functions (CDF, black dots) of the avalanche duration distributions. For visual comparison, the gray shading indicates the range (5–95%) of expected probabilities for the truncated power law with the same exponent as estimated from the experimental data and with the same number of samples (see Methods). P-values of truncated power law estimations (see Methods) are shown for each stimulus condition. We took the significance level to be 0.05, i.e., for *p* < 0.05 the power law hypothesis was rejected, whereas for *p* ≥ 0.05 the power law hypothesis was not rejected.
(PDF)

S7 Fig Average avalanche size scales with duration for the drifting grating stimulus condition. For each avalanche (solid purple dots) the size is plotted (log-log scale) vs the duration for each mouse. For each avalanche duration the average avalanche size (cyan dots) is plotted. The linear relationship on logarithmic axes reveals a power law relationship < *S* > ~ *D*^*β*^ between average avalanche size and duration as predicted by criticality theory. The fitted exponent *β* is derived from the linear regression line (yellow dashes). The predicted line (red) is derived from the predicted exponent *β* = (*α* − 1)/(*τ* − 1). Solid purple dots were included in the exponent estimation, open circles were not (see Methods).
(PDF)

S8 Fig Average avalanche size scales with duration for the natural movie stimulus condition. For each avalanche (solid purple dots) the size is plotted (log-log scale) vs the duration for each mouse. For each avalanche duration the average avalanche size (cyan dots) is plotted. The linear relationship on logarithmic axes reveals a power law relationship < *S* > ~ *D*^*β*^ between average avalanche size and duration as predicted by criticality theory. The fitted exponent *β* is derived from the linear regression line (yellow dashes). The predicted line (red) is derived from the predicted exponent *β* = (*α* − 1)/(*τ* − 1). Solid purple dots were included in the exponent estimation, open circles were not (see Methods).
(PDF)

S9 Fig Average avalanche size scales with duration for ongoing activity. For each avalanche (solid purple dots) the size is plotted (log-log scale) vs the duration for each mouse. For each avalanche duration the average avalanche size (cyan dots) is plotted. The linear relationship on logarithmic axes reveals a power law relationship < *S* > ~ *D*^*β*^ between average avalanche size and duration as predicted by criticality theory. The fitted exponent *β* is derived from the linear regression line (yellow dashes). The predicted line (red) is derived from the predicted exponent *β* = (*α* − 1)/(*τ* − 1). Solid purple dots were included in the exponent estimation, open circles were not (see Methods).
(PDF)

S10 Fig Irregular spiking is prevalent for all three stimulus conditions tested. The ISI coefficient of variation (CV_ISI_) distributions for the recorded neurons for 10 mice and three stimulus conditions. All CV distributions are widely distributed with most neurons having a CV_ISI_ larger than 1.
(PDF)

S11 Fig The zero-lag pairwise Pearson cross-correlation coefficients are broadly distributed for all stimulus conditions tested. The distributions of the zero-lag pairwise Pearson cross-correlation coefficients of the thresholded inferred spike probabilities for all mice and for the three stimulus conditions. Significance testing was obtained by comparing with uncorrelated spike trains of the same mean rate and adopting a p-value threshold of 0.1.
(PDF)

S12 Fig Changing the stimulus condition caused a redistribution of the pairwise cross-correlation coefficients among the pairs. TOP ROW: The cross-correlation coefficient matrix for the grating stimulus for each mouse, with the matrix clustered using a hierarchical clustering algorithm (see Methods). A subset of rows/columns are blank because neurons with noisy signal with no apparent calcium transient for a given stimulus condition were detected by visual inspection and excluded from further analysis for that stimulus condition. BOTTOM TWO ROWS: The cross-correlation coefficient matrix for the other two stimulus conditions (movie, ongoing), while maintaining the order of neurons as for grating stimulus (TOP ROW). This display illustrates the reorganization of the cross-correlation when varying the stimulus condition.
(PDF)
