# Supplementary figures and images for: Neocortical activity is stimulus- and scale-invariant
Source: PLoS One. 2017 May 10;12(5):e0177396. doi: 10.1371/journal.pone.0177396 (PMC5425225; doi:10.1371/journal.pone.0177396)

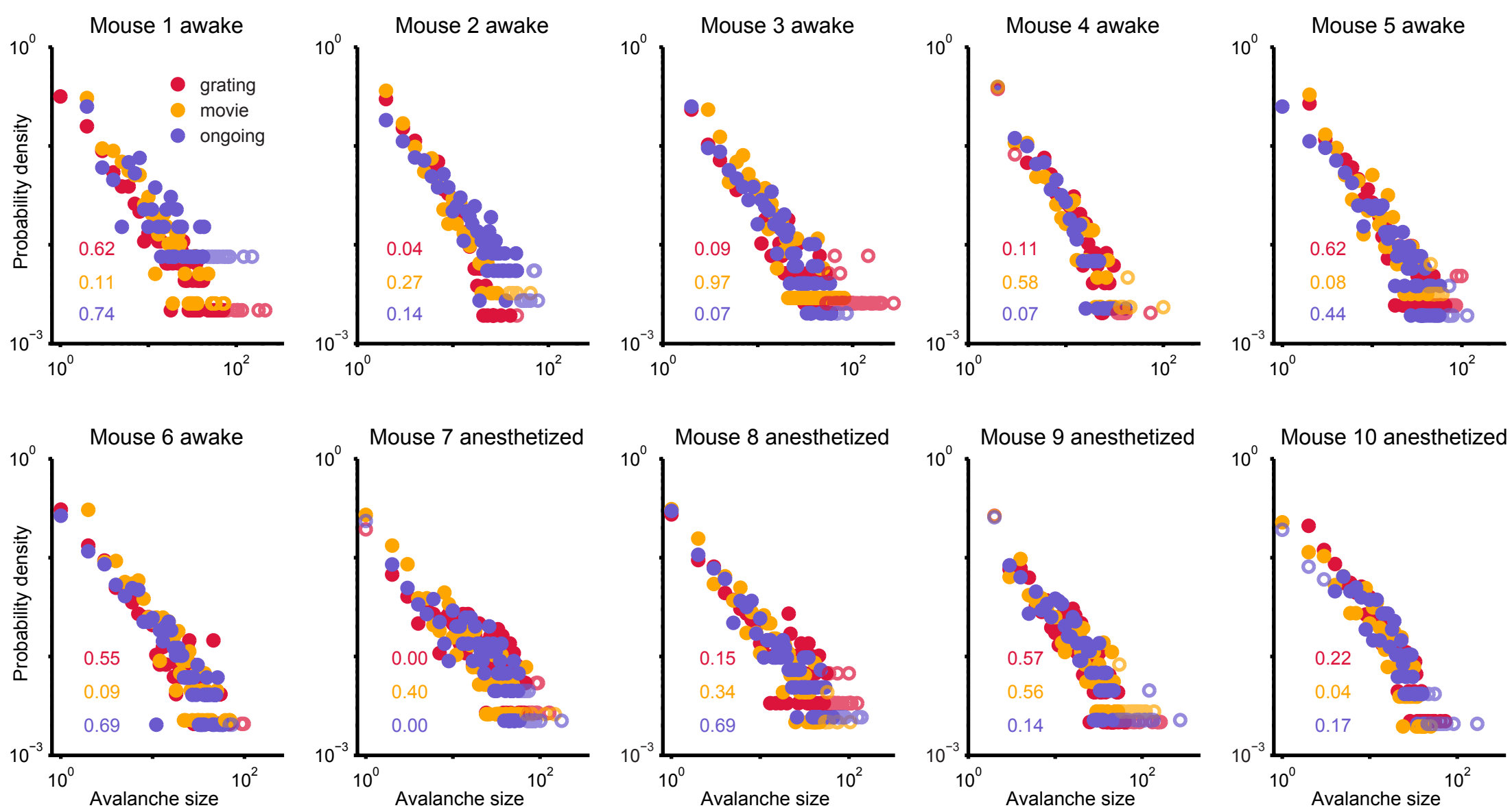

Supplement: S1 Fig — Probability density functions for avalanche sizes for each mouse and for the three stimulus conditions: grating (red), movie (yellow), and ongoing (blue). The solid dots denote the avalanches included for fitting to a truncated power law; open circles denote avalanches that were excluded in the fitting procedure (see Methods). P-values of truncated power law estimations (see Methods) are shown for each stimulus condition (color assignment as in legend). We took the significance level to be 0.05, i.e., for p < 0.05 the power law hypothesis was rejected, whereas for p ≥ 0.05 the power law hypothesis was not rejected. (PDF) [file pone.0177396.s001.pdf]

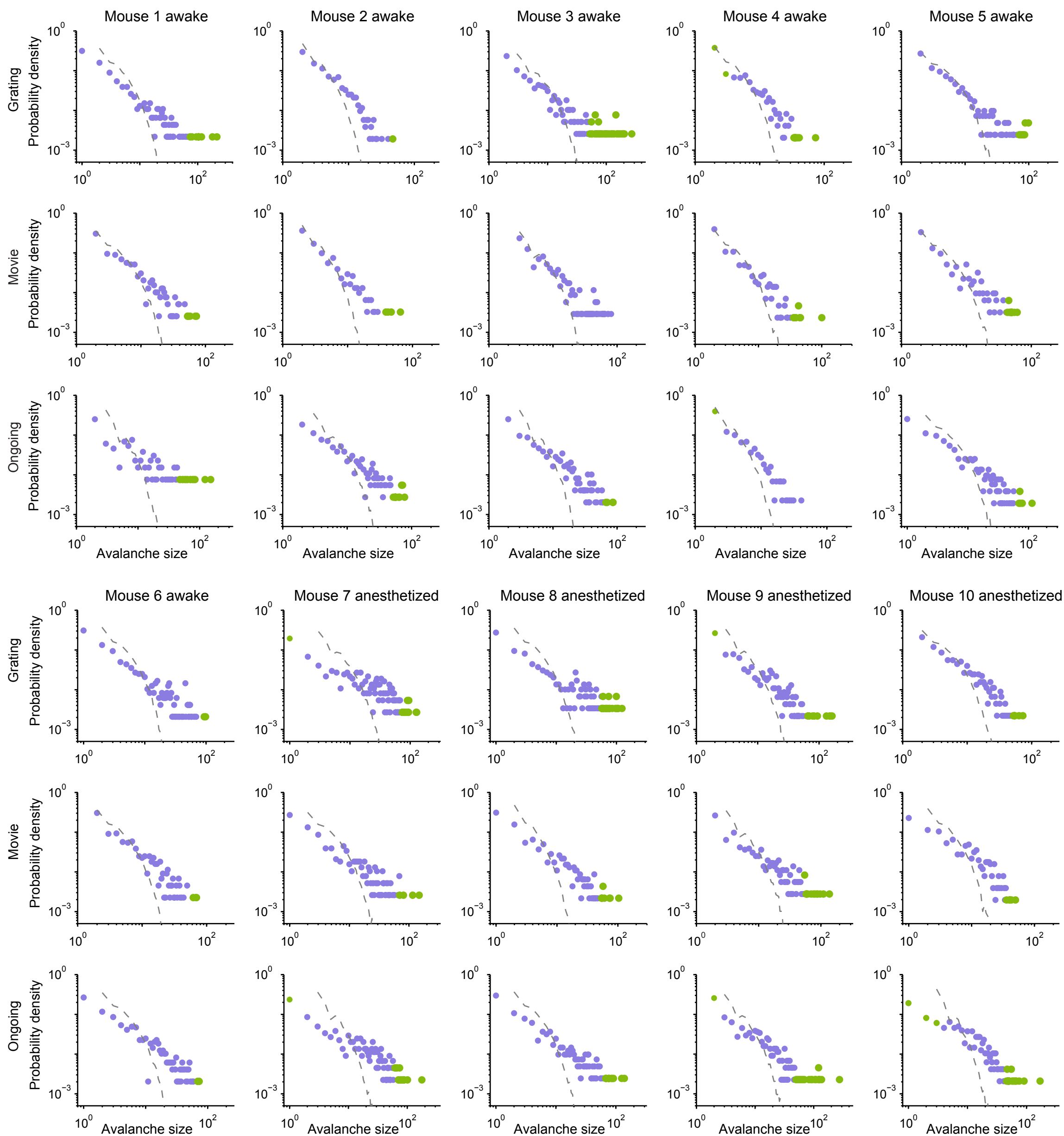

Supplement: S2 Fig — The violet dots denote the avalanches (based on recorded spike trains) that were included for fitting to a truncated power law; pale green dots denote avalanches that were excluded in the fitting procedure (see Methods). Shuffling spike times abolishes large avalanches and results in avalanche size distributions (dashed gray lines) that are inconsistent with a power law. (PDF) [file pone.0177396.s002.pdf]

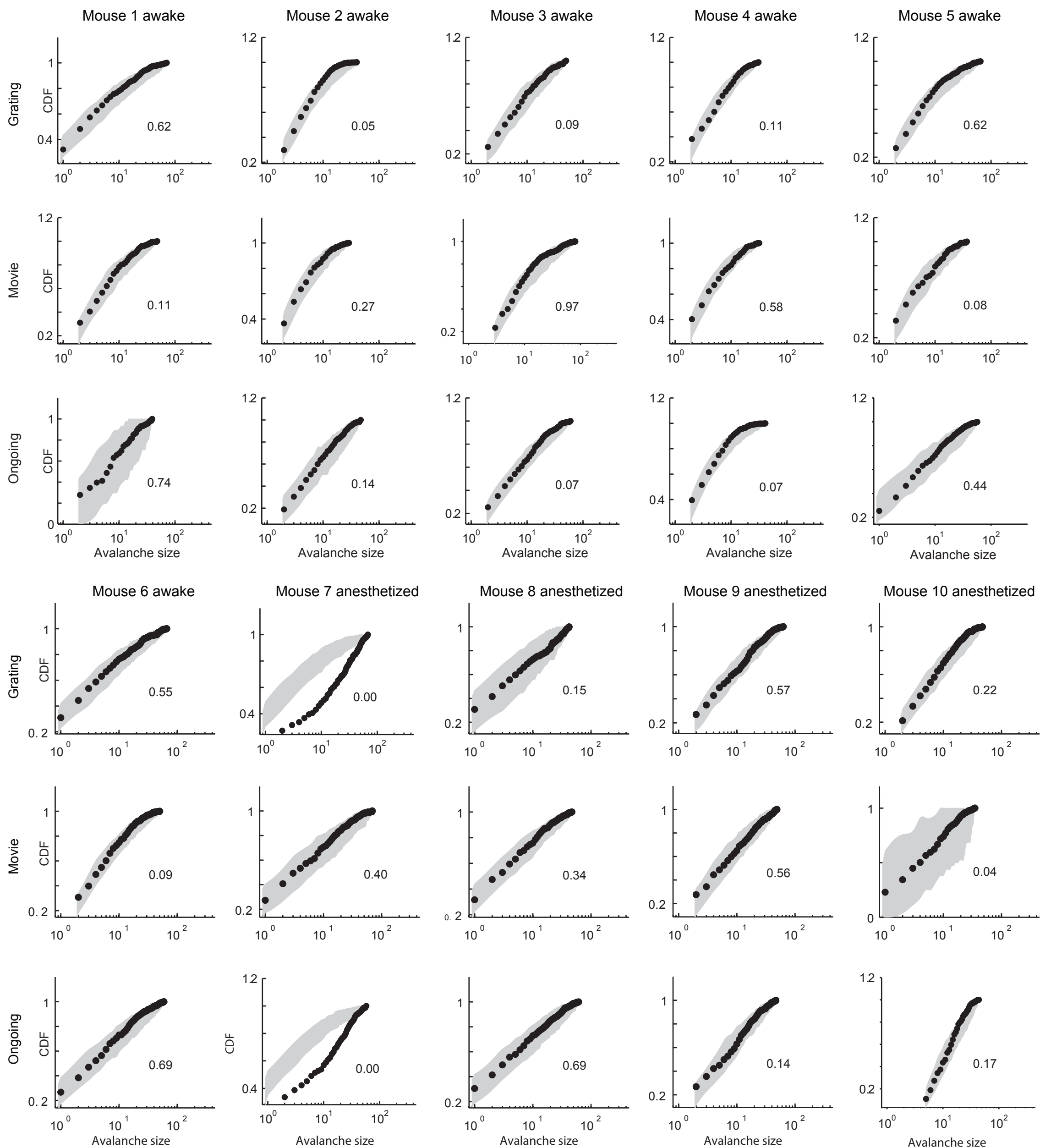

Supplement: S3 Fig — For visual comparison, the gray shading indicates the range (5–95%) of expected probabilities for the truncated power law with the same exponent as estimated from the experimental data and with the same number of samples (see Methods). P-values of truncated power law estimations (see Methods) are shown for each stimulus condition. We took the significance level to be 0.05, i.e., for p < 0.05 the power law hypothesis was rejected, whereas for p ≥ 0.05 the power law hypothesis was not rejected. (PDF) [file pone.0177396.s003.pdf]

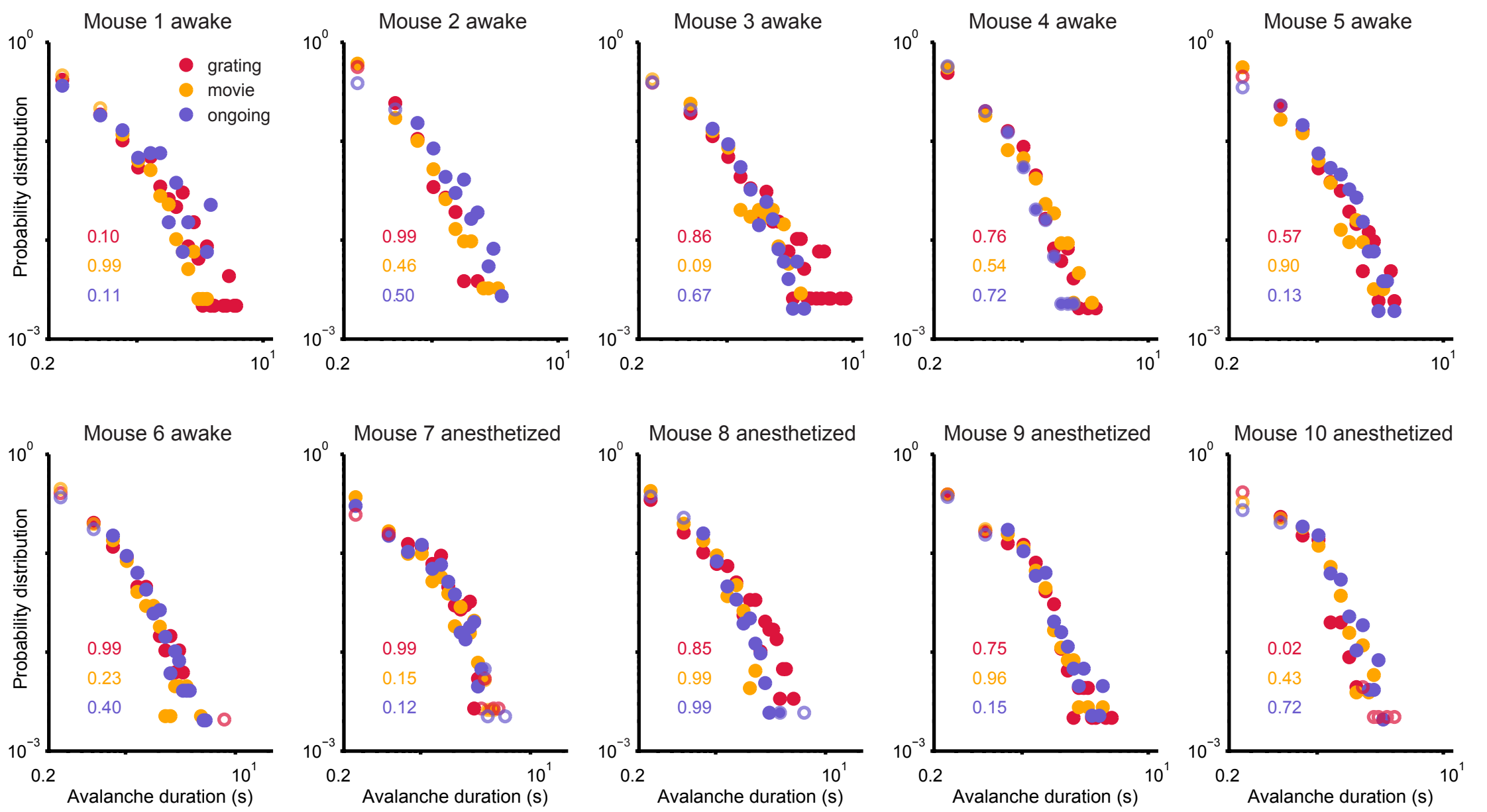

Supplement: S4 Fig — Probability density functions for avalanche durations for each mouse and for the three stimulus conditions: grating (red), movie (yellow), and ongoing (blue). The solid dots denote the avalanches included for fitting to a truncated power law; open circles denote avalanches that were excluded in the fitting procedure (see Methods). P-values of truncated power law estimations (see Methods) are shown for each stimulus condition (color assignment as in legend). We took the significance level to be 0.05, i.e., for p < 0.05 the power law hypothesis was rejected, whereas for p ≥ 0.05 the power law hypothesis was not rejected. (PDF) [file pone.0177396.s004.pdf]

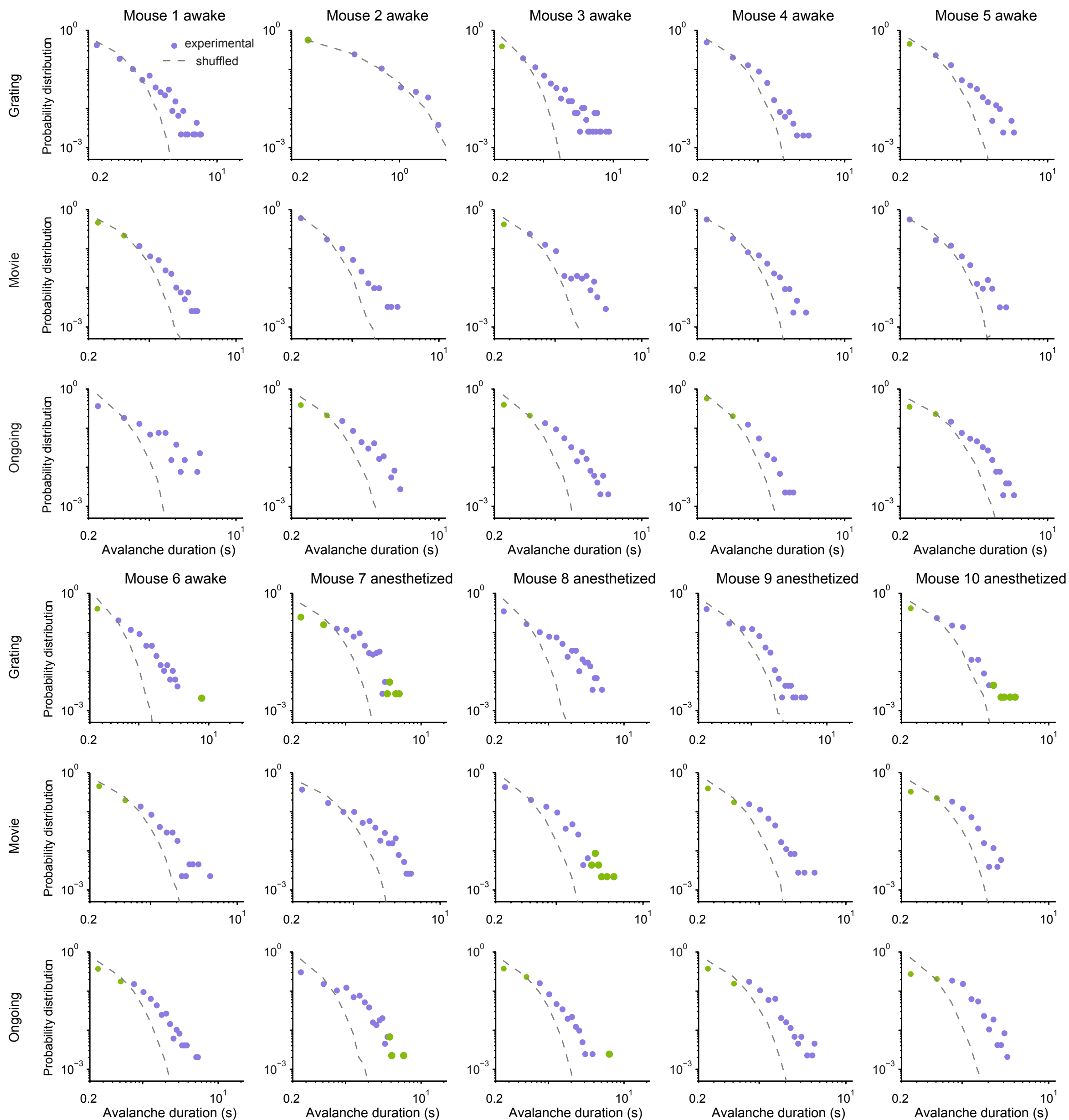

Supplement: S5 Fig — The violet dots denote the avalanches (based on recorded spike trains) that were included for fitting to a truncated power law; pale green dots denote avalanches that were excluded in the fitting procedure (see Methods). Shuffling spike times abolishes long duration avalanches and results in avalanche duration distributions (dashed gray lines) that are inconsistent with a power law. (PDF) [file pone.0177396.s005.pdf]

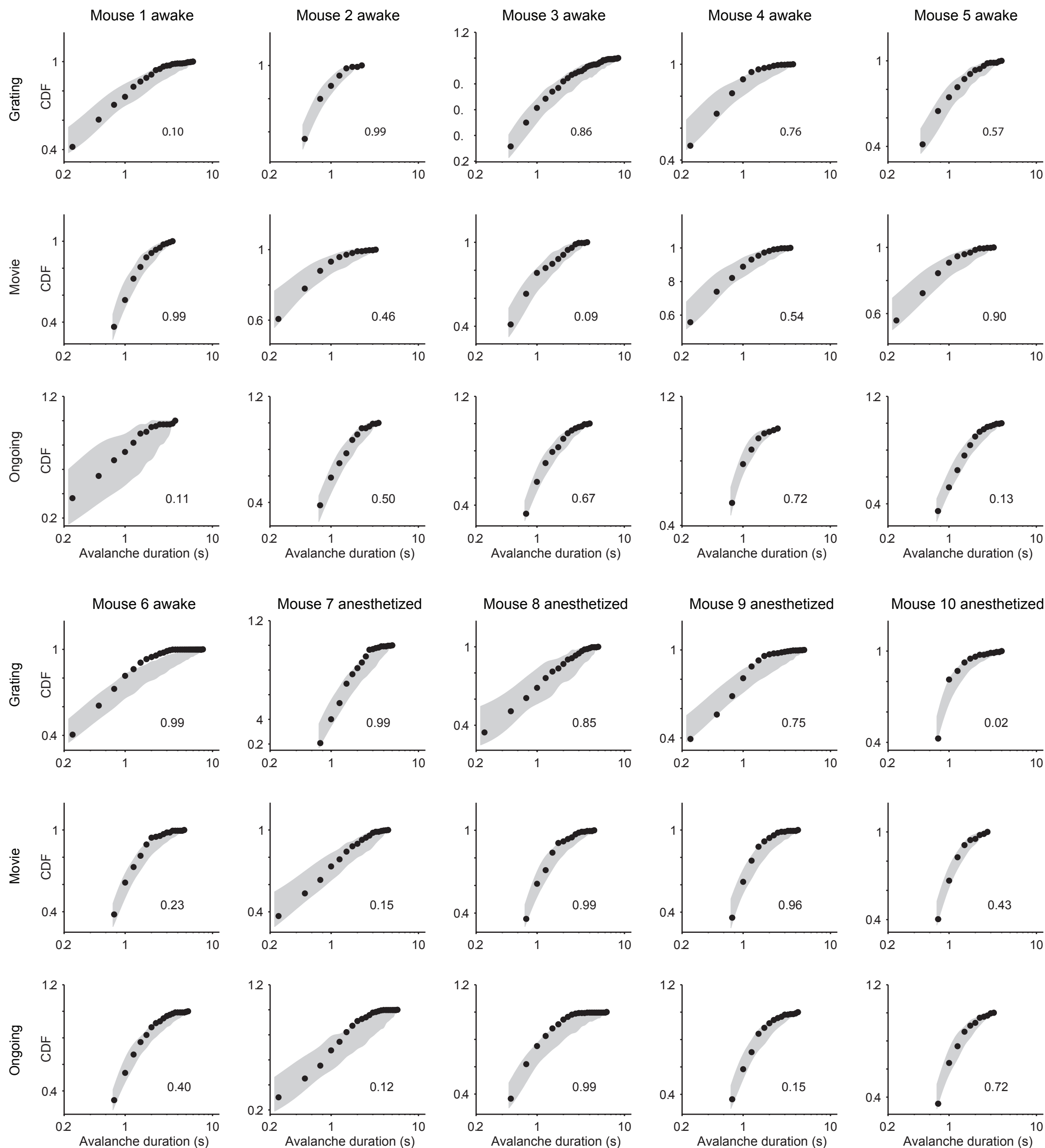

Supplement: S6 Fig — For visual comparison, the gray shading indicates the range (5–95%) of expected probabilities for the truncated power law with the same exponent as estimated from the experimental data and with the same number of samples (see Methods). P-values of truncated power law estimations (see Methods) are shown for each stimulus condition. We took the significance level to be 0.05, i.e., for p < 0.05 the power law hypothesis was rejected, whereas for p ≥ 0.05 the power law hypothesis was not rejected. (PDF) [file pone.0177396.s006.pdf]

○ real excluded    ● real    — predicted    — fitted    ● <S> vs D

### Grating

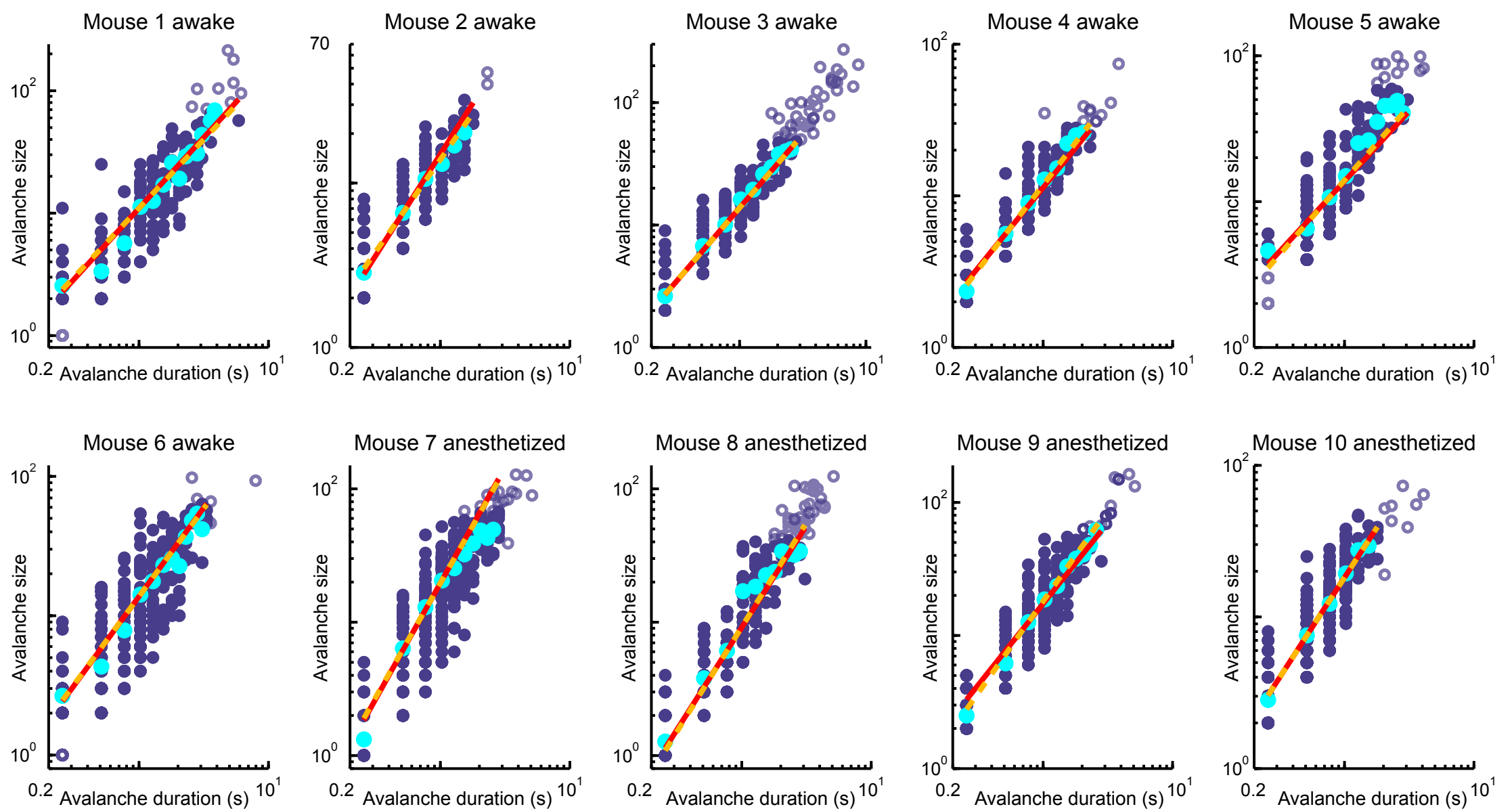

Supplement: S7 Fig — For each avalanche (solid purple dots) the size is plotted (log-log scale) vs the duration for each mouse. For each avalanche duration the average avalanche size (cyan dots) is plotted. The linear relationship on logarithmic axes reveals a power law relationship < S > ~ Dβ between average avalanche size and duration as predicted by criticality theory. The fitted exponent β is derived from the linear regression line (yellow dashes). The predicted line (red) is derived from the predicted exponent β = (α − 1)/(τ − 1). Solid purple dots were included in the exponent estimation, open circles were not (see Methods). (PDF) [file pone.0177396.s007.pdf]

# Movie

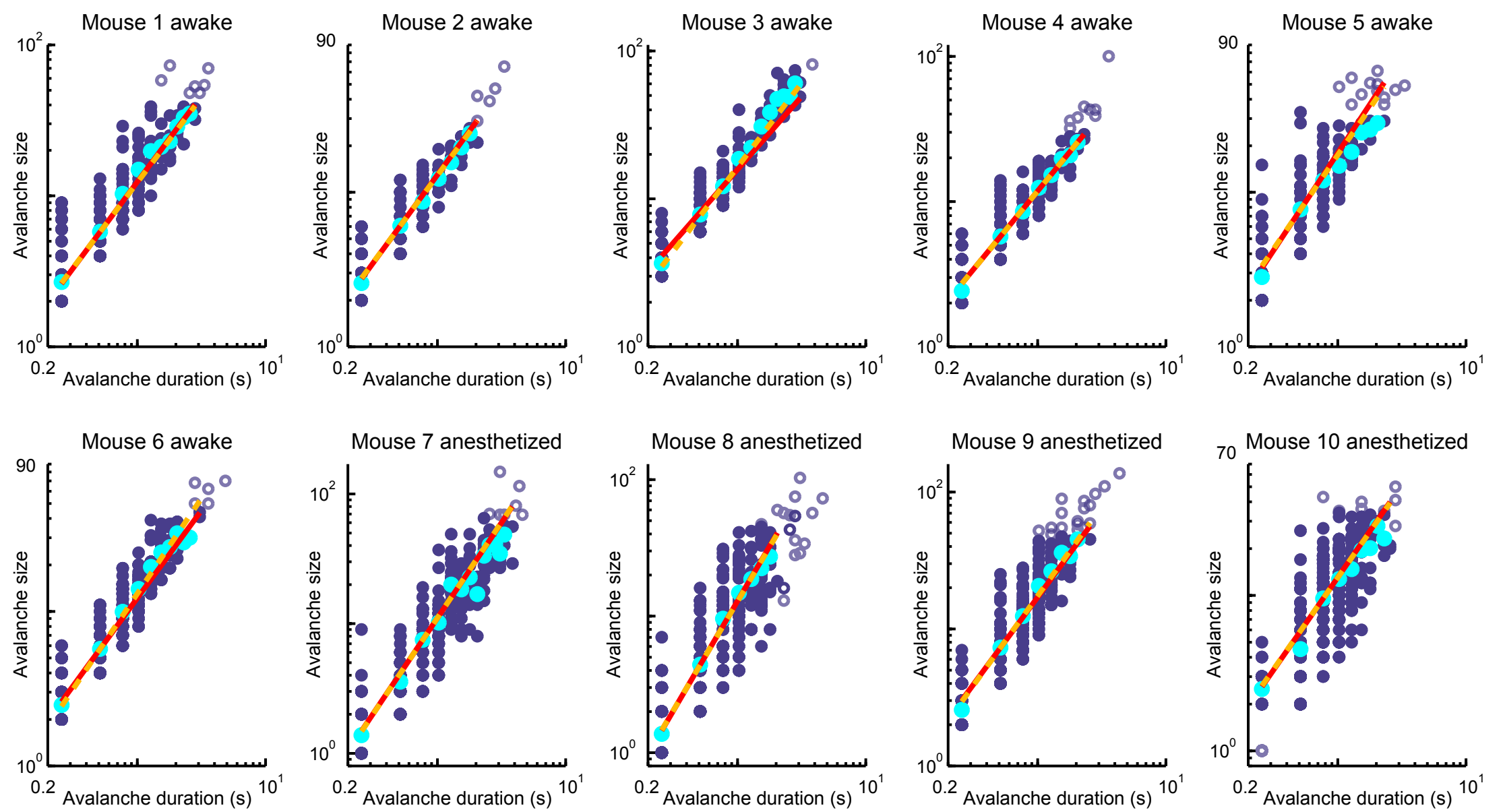

Supplement: S8 Fig — For each avalanche (solid purple dots) the size is plotted (log-log scale) vs the duration for each mouse. For each avalanche duration the average avalanche size (cyan dots) is plotted. The linear relationship on logarithmic axes reveals a power law relationship < S > ~ Dβ between average avalanche size and duration as predicted by criticality theory. The fitted exponent β is derived from the linear regression line (yellow dashes). The predicted line (red) is derived from the predicted exponent β = (α − 1)/(τ − 1). Solid purple dots were included in the exponent estimation, open circles were not (see Methods). (PDF) [file pone.0177396.s008.pdf]

### Ongoing

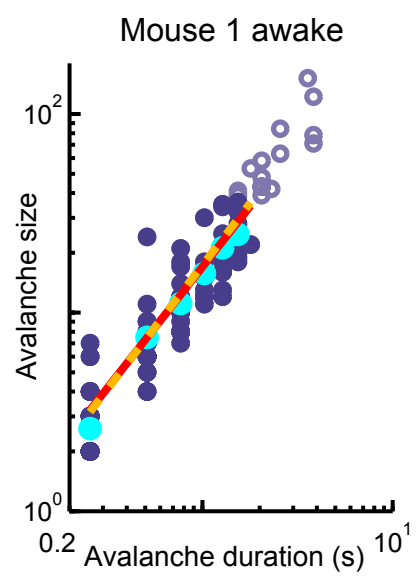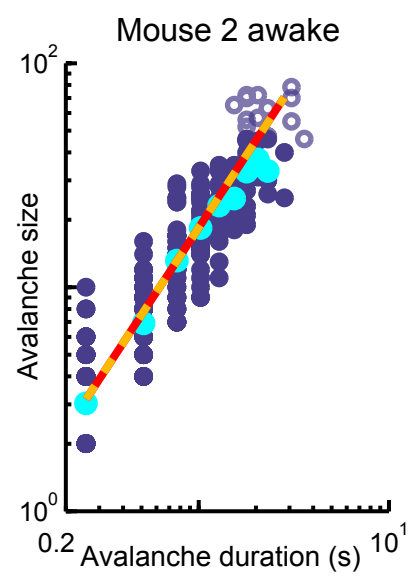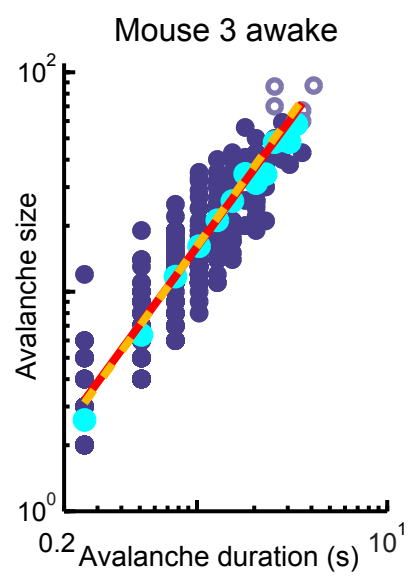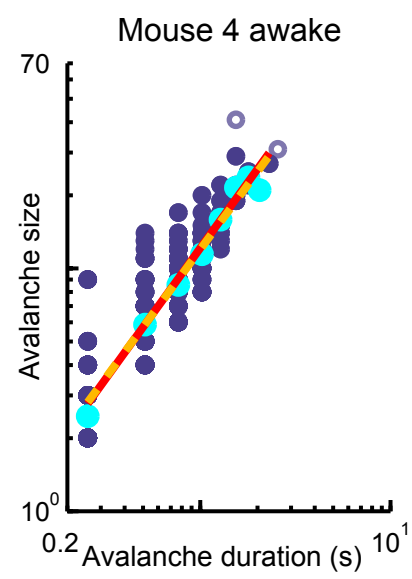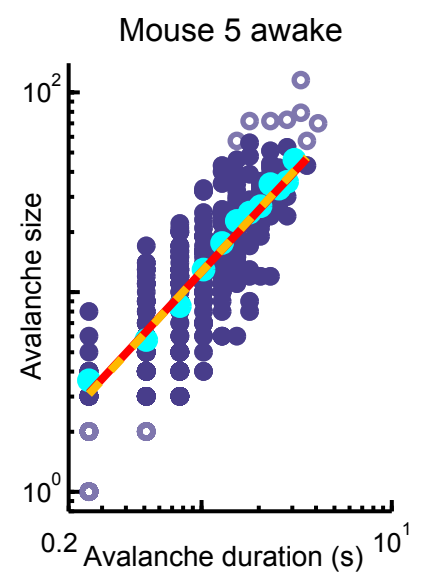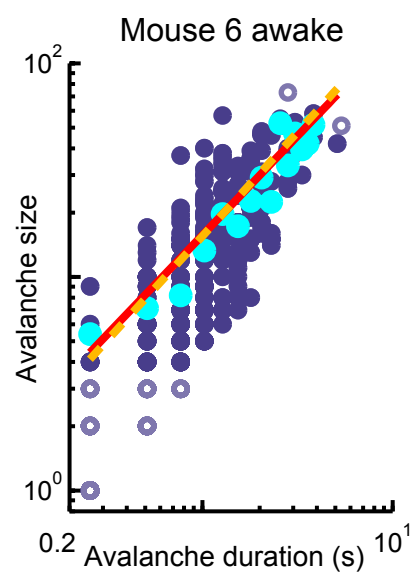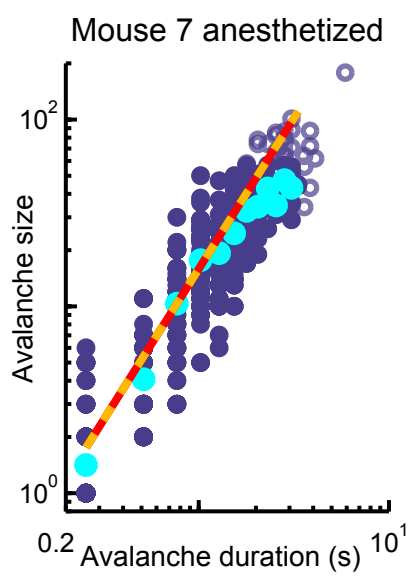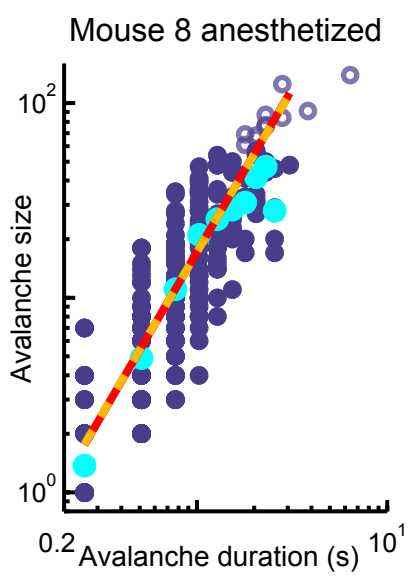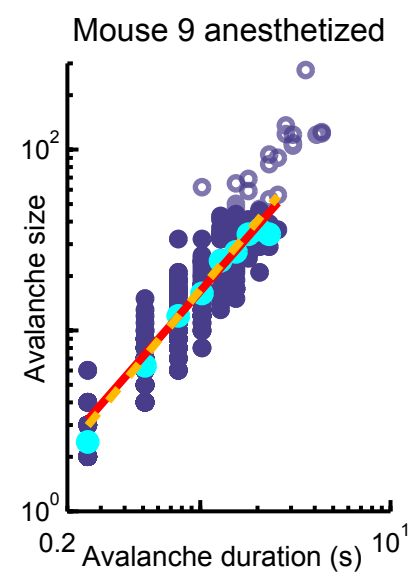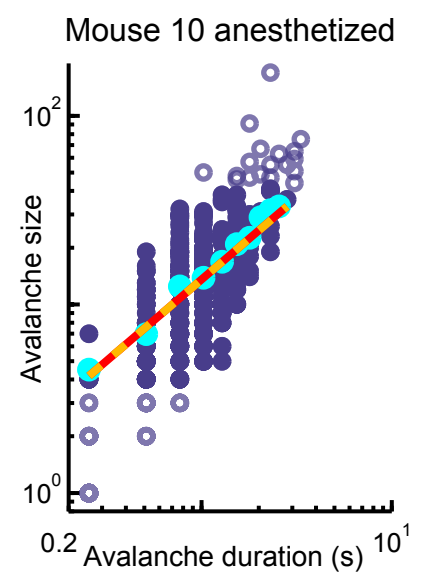

Supplement: S9 Fig — For each avalanche (solid purple dots) the size is plotted (log-log scale) vs the duration for each mouse. For each avalanche duration the average avalanche size (cyan dots) is plotted. The linear relationship on logarithmic axes reveals a power law relationship < S > ~ Dβ between average avalanche size and duration as predicted by criticality theory. The fitted exponent β is derived from the linear regression line (yellow dashes). The predicted line (red) is derived from the predicted exponent β = (α − 1)/(τ − 1). Solid purple dots were included in the exponent estimation, open circles were not (see Methods). (PDF) [file pone.0177396.s009.pdf]

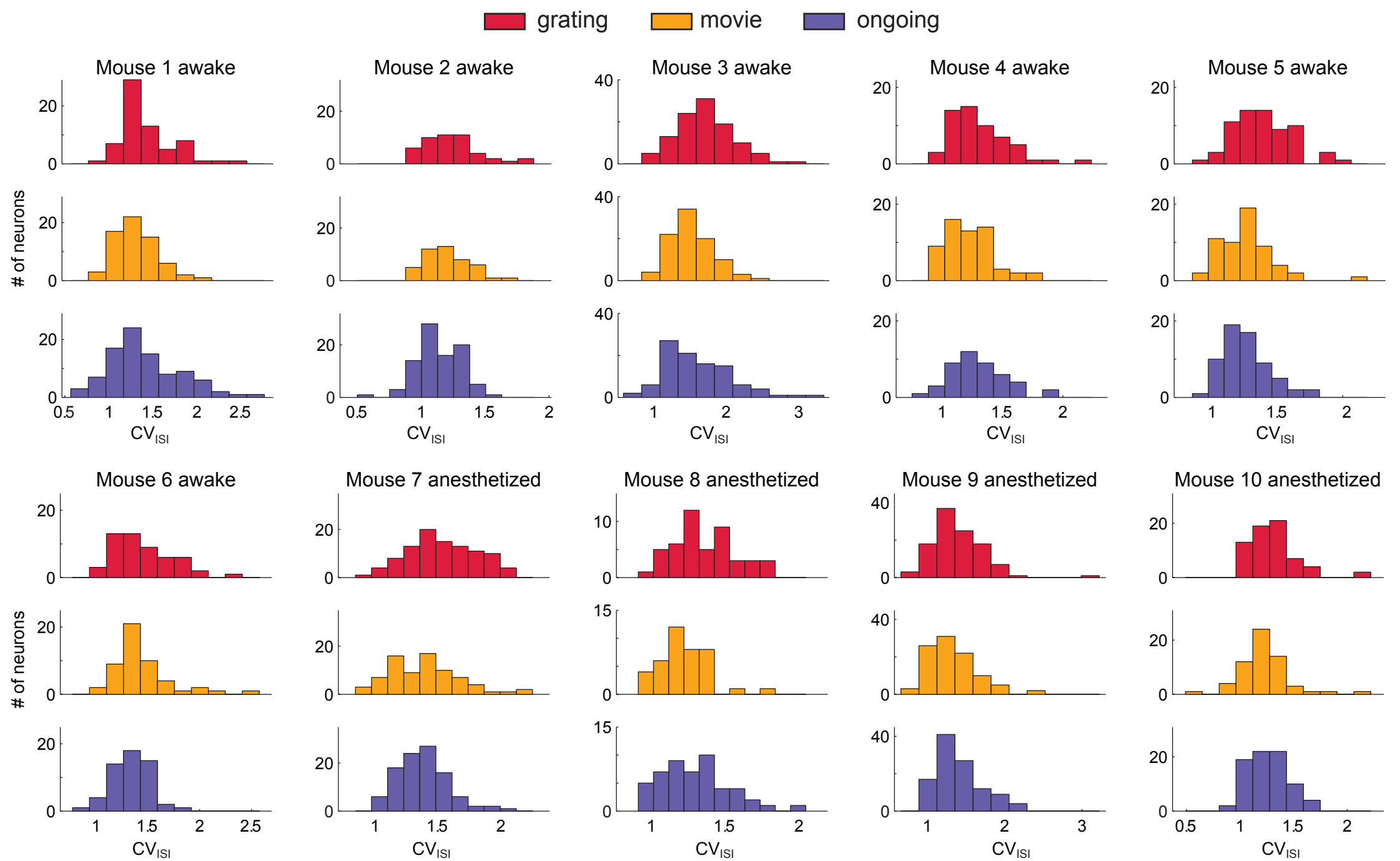

Supplement: S10 Fig — The ISI coefficient of variation (CVISI) distributions for the recorded neurons for 10 mice and three stimulus conditions. All CV distributions are widely distributed with most neurons having a CVISI larger than 1. (PDF) [file pone.0177396.s010.pdf]

■ ■ ■ significant coefficients      □ □ □ insignificant coefficients

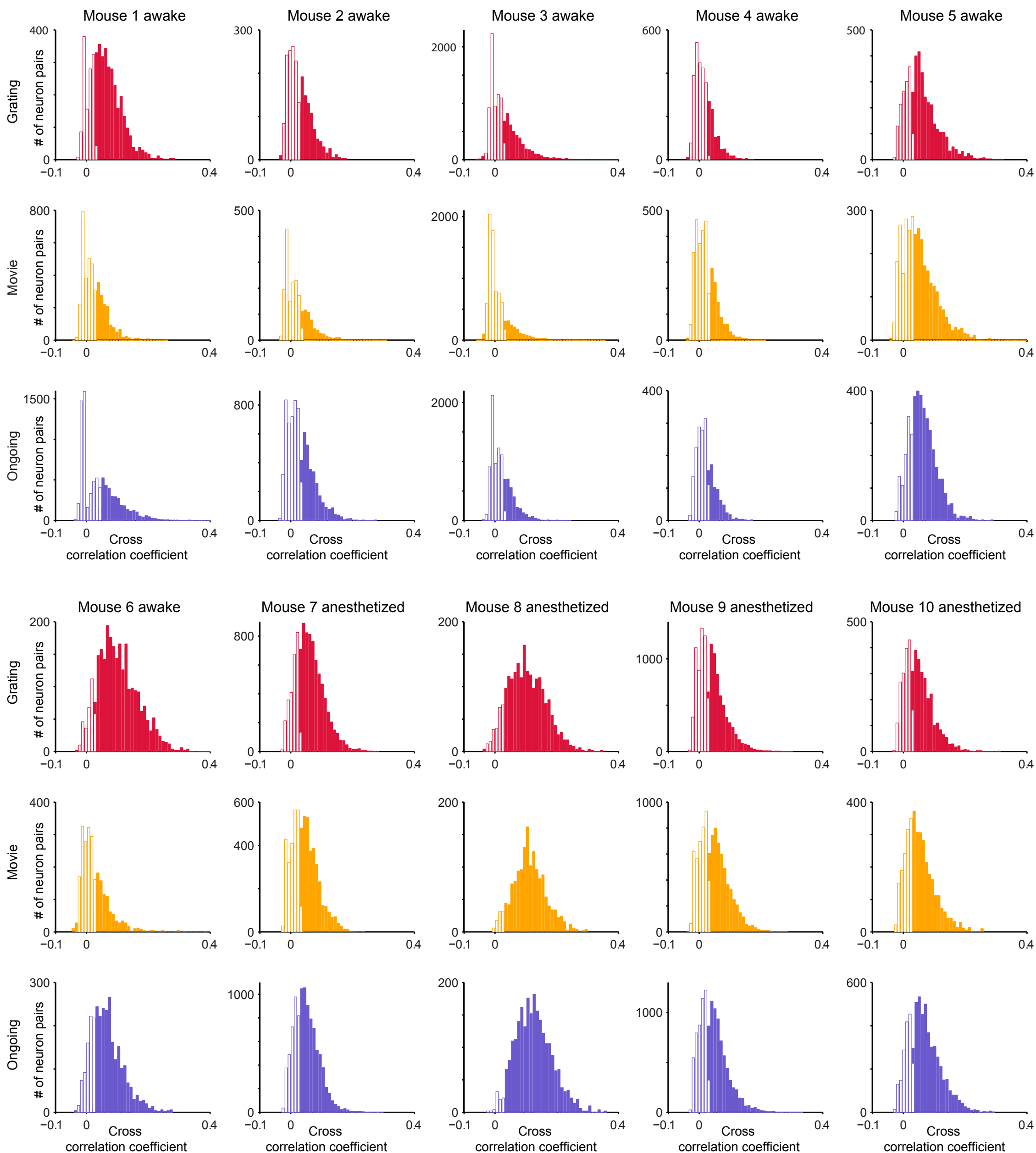

Supplement: S11 Fig — The distributions of the zero-lag pairwise Pearson cross-correlation coefficients of the thresholded inferred spike probabilities for all mice and for the three stimulus conditions. Significance testing was obtained by comparing with uncorrelated spike trains of the same mean rate and adopting a p-value threshold of 0.1. (PDF) [file pone.0177396.s011.pdf]

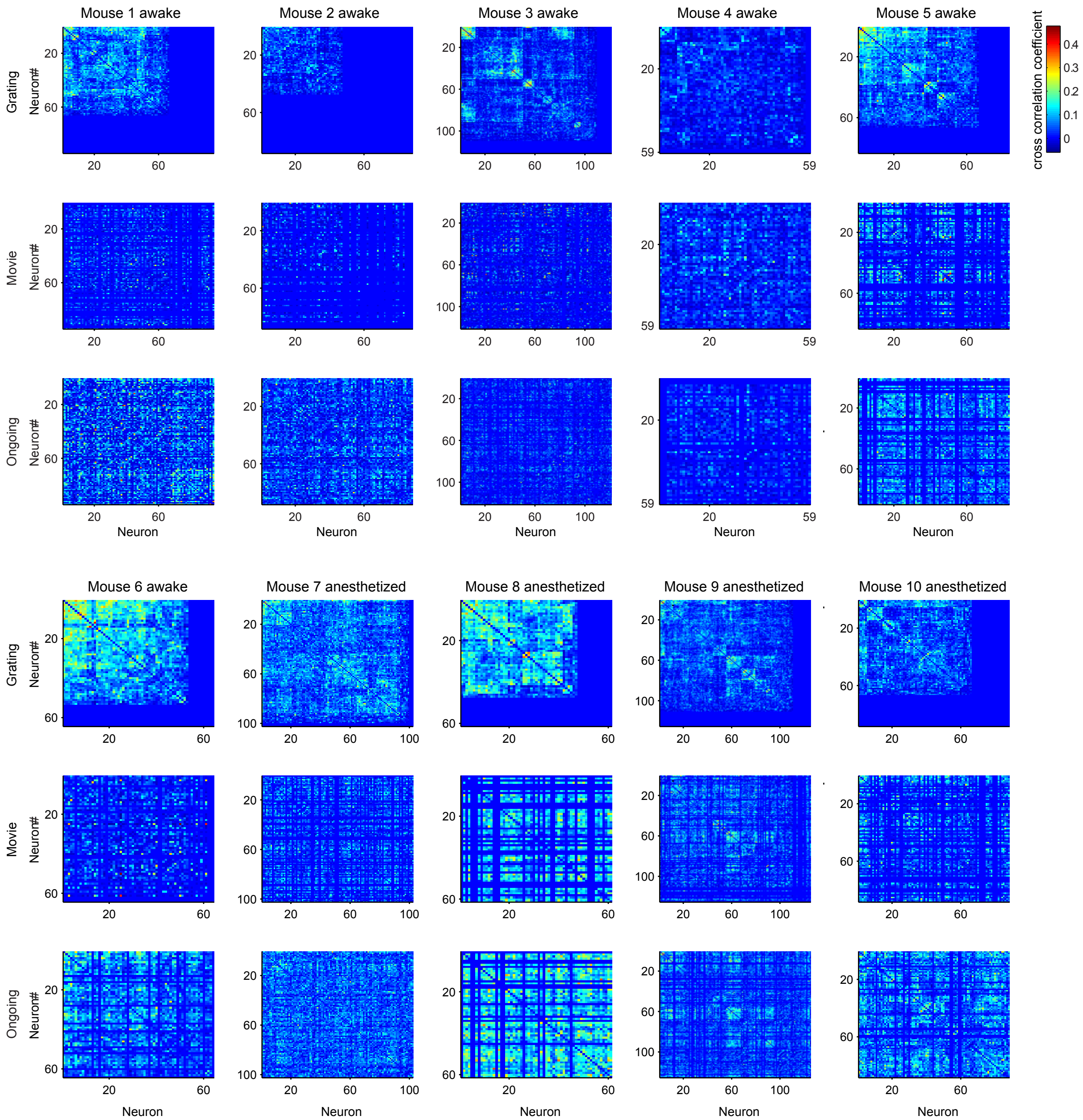

Supplement: S12 Fig — TOP ROW: The cross-correlation coefficient matrix for the grating stimulus for each mouse, with the matrix clustered using a hierarchical clustering algorithm (see Methods). A subset of rows/columns are blank because neurons with noisy signal with no apparent calcium transient for a given stimulus condition were detected by visual inspection and excluded from further analysis for that stimulus condition. BOTTOM TWO ROWS: The cross-correlation coefficient matrix for the other two stimulus conditions (movie, ongoing), while maintaining the order of neurons as for grating stimulus (TOP ROW). This display illustrates the reorganization of the cross-correlation when varying the stimulus condition. (PDF) [file pone.0177396.s012.pdf]
